# Photochemical strategies in divalent samarium catalysis

**DOI:** 10.3762/bjoc.22.105

**Published:** 2026-09-25

**Authors:** Takahito Kuribara, Tetsuhiro Nemoto

**Affiliations:** 1 Graduate School of Pharmaceutical Sciences, Chiba University, 1-8-1, Inohana, Chuo-ku, Chiba 260-8675, Japan

**Keywords:** antenna ligand, catalysis, reduction, samarium, visible light

## Abstract

Divalent samarium reagents, particularly samarium diiodide (SmI_2_), have long been recognized as the most powerful and versatile single-electron reductants in organic synthesis. Several catalytic strategies have been developed for Sm(II) chemistry over the past three decades, including approaches based on metal co-reductants, electrochemical reduction, and radical relay mechanisms. Photochemical strategies have recently emerged as new platforms for catalytic Sm(II) chemistry, leading to the development of a broad range of reductive transformations. This review highlights the recent progress in the photochemical generation of Sm(II) species and their applications in organic synthesis.

## Introduction

Samarium(II) diiodide (SmI_2_) is an important single-electron reducing agent used in organic synthesis [1–4]. Since Kagan et al. reported its practical preparation and synthetic applications [5–6], SmI_2_ has been used widely in diverse reductive transformations, including the reduction of carbonyl compounds and organic halides, pinacol coupling, ketyl–olefin coupling, and radical cyclization [1–4]. SmI_2_ exhibits unique reactivity and selectivity depending on the reaction conditions such as the choice of ligand, solvent, and proton source [7–9]. Moreover, its ability to generate radical intermediates under mild reaction conditions, and its excellent functional group tolerance, has made SmI_2_ a promising reagent for the synthesis of natural products and biologically active molecules [10–13].

**Figure 1:**
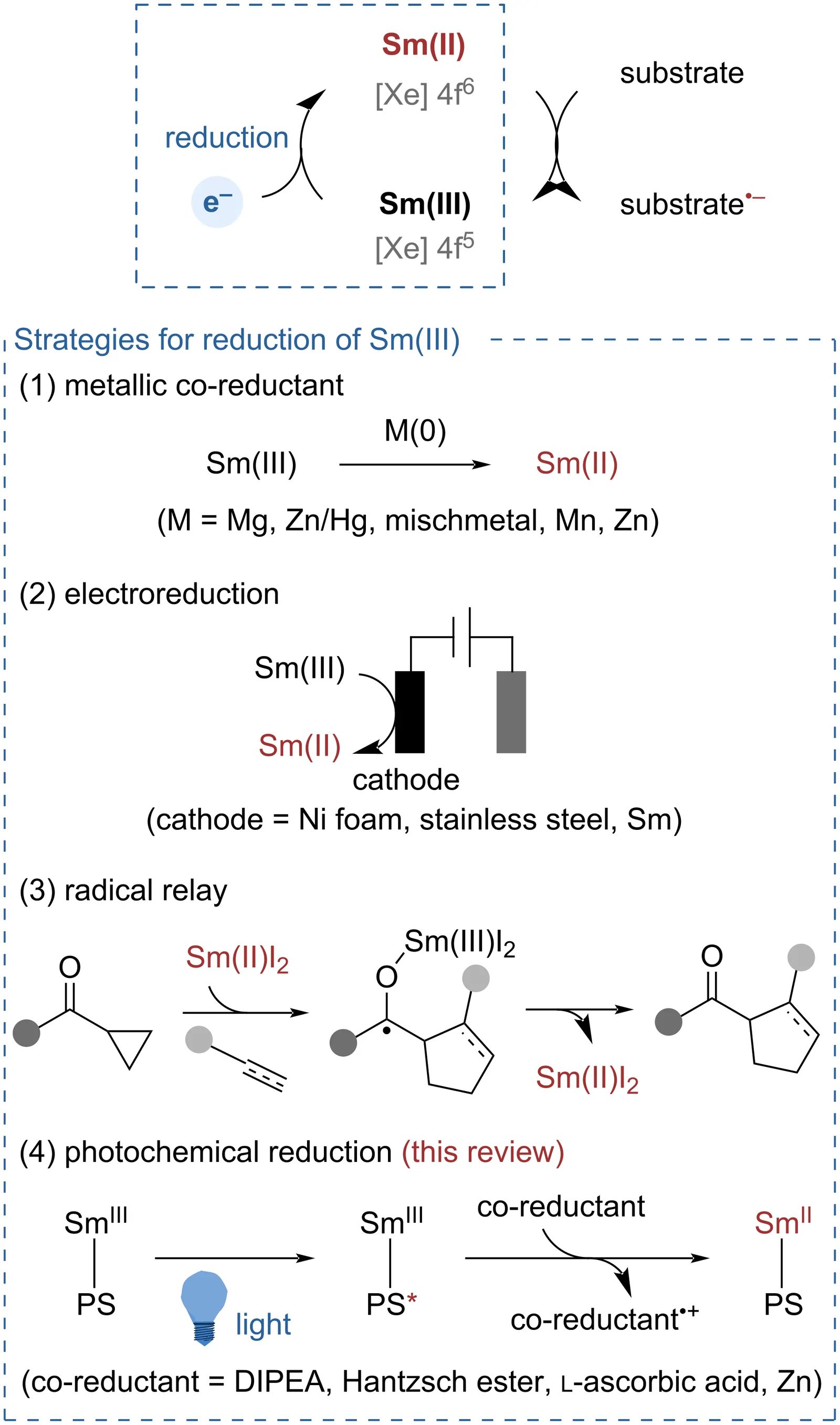
Strategies for catalytic Sm(II) chemistry. PS = photosensitizer.

However, most SmI_2_-mediated reactions require stoichiometric quantities of reagents or an excess [14]. The standard reduction potential for the reduction of Sm(III) to Sm(II) is −1.55 V vs SHE (standard hydrogen electrode), which is highly negative [14]. Moreover, Sm(III) often forms strong Sm–O bonds with alkoxides derived from carbonyl compounds or proton donors, making regeneration of the active Sm(II) species difficult [15]. Therefore, achieving catalytic turnover through the Sm(III)/Sm(II) redox cycle has long been challenging. Several strategies have been developed to address this challenge (Figure 1). These include (1) the use of metallic co-reductants such as magnesium, Zn/Hg, mischmetal, manganese, and zinc [15–23], (2) the electrochemical reduction of Sm(III) [15,24–38], and (3) redox-neutral catalytic reactions based on the radical relay mechanism [39–44]. These approaches demonstrated the feasibility of catalytic Sm(II) chemistry. However, several limitations are extant, including the requirement of preformed Sm(II) reagents, high catalyst loadings (typically 10–20 mol %), the use of excess metallic reductants or additives, and a limited reaction scope. Photochemical strategies have recently emerged as approaches to overcome these limitations and have attracted considerable attention.

**Figure 2:**
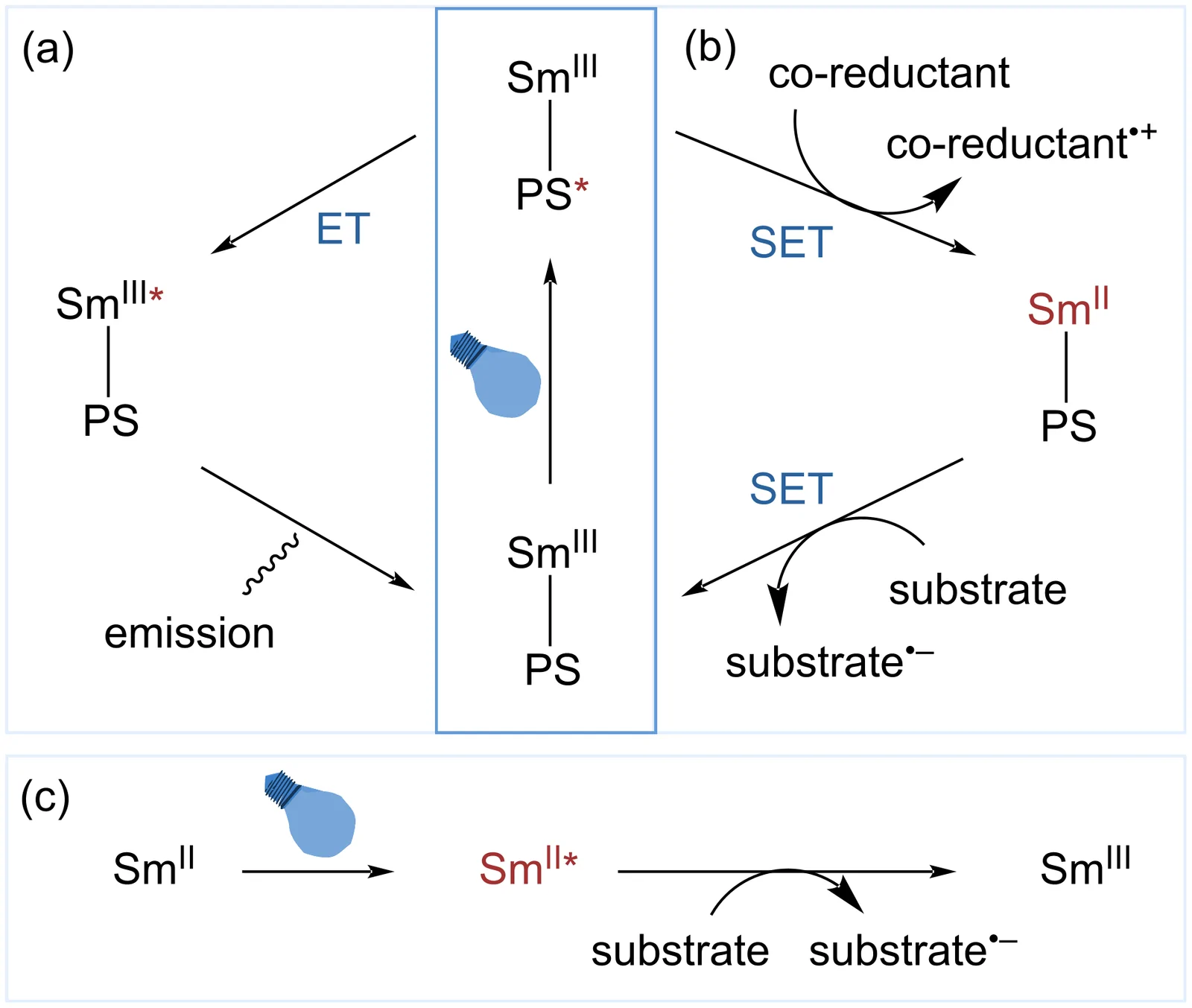
Photochemical activation of samarium species. (a) Energy transfer (ET) from a photosensitizer (PS) to Sm(III). (b) Single-electron transfer (SET) to Sm(III) in the presence of a co-reductant, generating Sm(II) in situ. (c) Direct photoexcitation of Sm(II).

Conventionally, the photochemistry of lanthanoids, including samarium, has developed primarily in the field of luminescence [45–48]. Excited-state Ln(III) species exhibit characteristic long-lived narrowband emissions, which have been widely utilized in luminescent materials and sensing applications. In general, Ln(III) exhibits extremely weak light absorption because of Laporte-forbidden 4f–4f transitions. Therefore, light-absorbing organic ligands, known as antenna ligands, have been introduced to efficiently absorb light – typically in the UV region – and transfer the resulting triplet energy to the coordinated Ln(III) center through the antenna effect (Figure 2a). In addition, divalent lanthanoids such as Sm(II), Yb(II), and Eu(II) undergo 4f→5d excitation to generate highly reducing excited states, enabling their application in photoreductive transformations (Figure 2c) [49–63]. However, the use of light energy to reduce Ln(III), thereby generating catalytically active reducing Ln(II) species, remains largely unexplored (Figure 2b) [64]. Therefore, the development of efficient photochemical methods for Sm(III) reduction is expected to provide new catalytic strategies based on Sm(III)/Sm(II) redox cycles.

Recently, considerable effort has been expended toward achieving the catalytic generation of reactive Sm(II) species from stable Sm(III) precursors using visible-light energy as the driving force [65–73]. The development of photoactive samarium complexes [65–69], redox-active antenna ligands [70–71], and dual catalytic systems combining samarium catalysts with external photoredox catalysts [72–73] has enabled photo-driven Sm(II)-catalyzed reduction reactions with mild sacrificial co-reductants. Furthermore, beyond the catalytic regeneration of Sm(II), these photochemical strategies enable the use of light energy, mild reaction conditions, and precise control of the coordination environment of samarium, thereby allowing the fine-tuning of reactivity and selectivity [65–73]. Consequently, they have facilitated challenging transformations, including the activation of difficult-to-reduce substrates [65–73], multielectron reduction [68–69], integration of Sm(II) catalysis with photooxidation and Lewis acid catalysis [70–71], and catalytic asymmetric reactions [73]. Moreover, the use of visible light as a sustainable energy source makes this strategy environmentally benign.

This review focuses on the rapid development of photochemical strategies for Sm(II) catalysis since 2023. Comprehensive reviews on catalytic Sm(II) chemistry were published by Maity in 2021 [74], Wei [75], and Procter [14] in 2025. Electrochemical approaches were reviewed by Lundberg in 2023 [76]. A comprehensive review on organolanthanoid chemistry was reported by Simler and Nocton in 2023 [77]. Unlike these previous reviews, we herein highlight photochemical Sm(III)/Sm(II) redox chemistry, with particular emphasis on catalyst design, catalytic mechanisms, and applications in organic synthesis. Finally, we discuss the key features of these photochemical strategies and provide perspectives for the future development of photochemical strategies in catalytic Sm(II) chemistry.

## Review

### Coumarin-based chromophores

**Figure 3:**
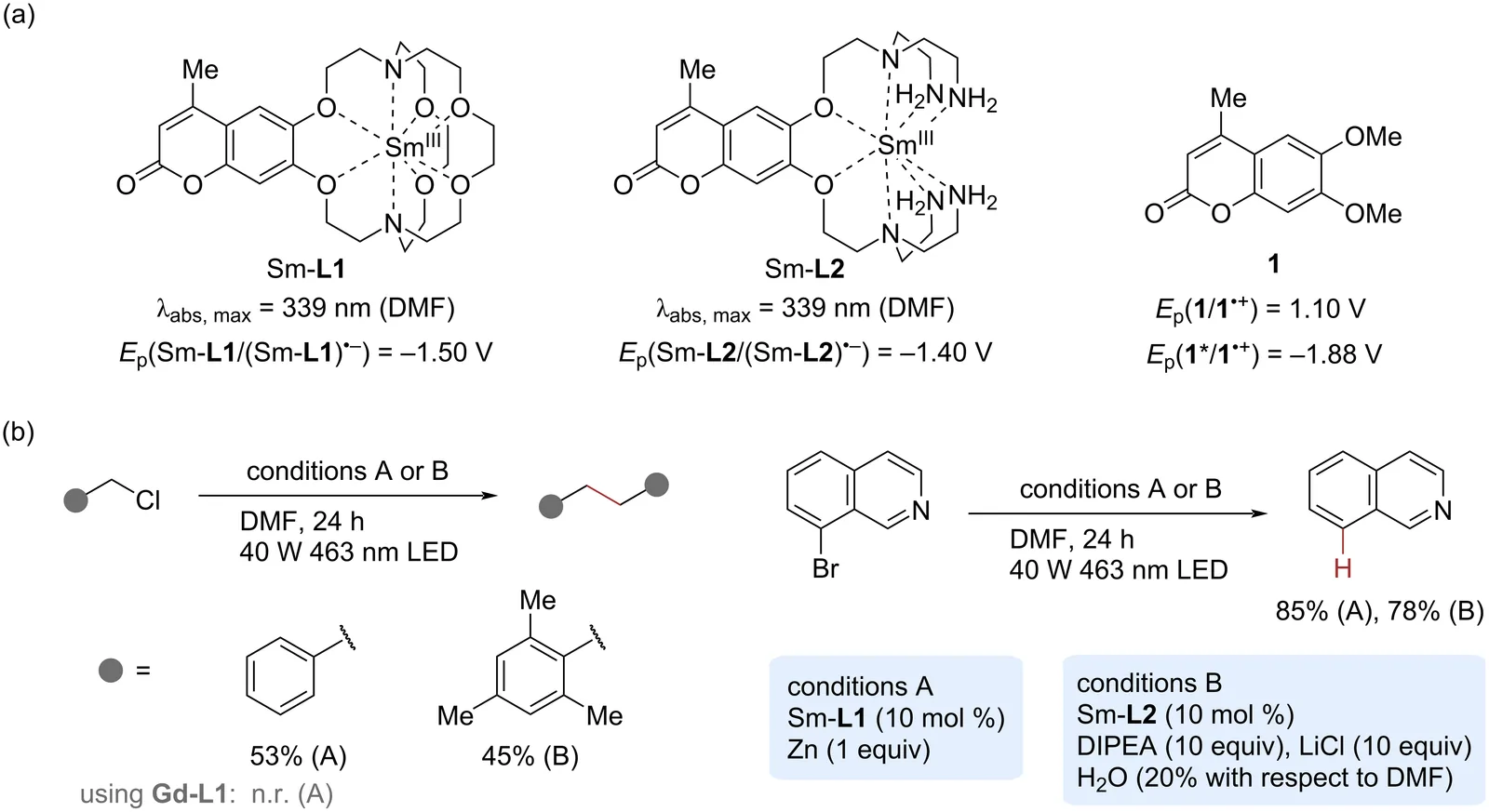
Early examples of samarium-catalyzed photoreactions based on coumarin chromophores [65]. (a) Structures of the samarium complexes. Potentials are given vs Fc/Fc^+^ in DMF. (b) Selected examples of samarium-catalyzed reduction reactions.

In 2023, Borbas et al. reported the photocatalytic generation of Eu(II) and Sm(II) reductants from trivalent precursors and highlighted their application in organic synthesis [65]. Although this study focused on europium complexes, several reactions using samarium complexes were also investigated. The authors designed stable Sm(III) complexes incorporating a 6,7-dioxycoumarin chromophore bearing a cryptand-type ligand **L1** or an open-chain amine ligand **L2** (Figure 3a). Both Sm-**L1** and Sm-**L2** exhibited absorption maxima at 339 nm, with absorption bands extending into the visible region. Furthermore, based on the oxidation potential of 6,7-dimethoxycoumarin **1** (*E*_p_(**1**/**1**^•+^) = +1.10 V vs Fc/Fc^+^) and its emission wavelength (λ_em_ = 415 nm), the excited-state reduction potential was estimated as being −1.88 V (*E*_p_(1*/**1**^•+^) vs Fc/Fc^+^). This value is sufficiently more negative than the reduction potentials of Sm-**L1** (*E*_p_(Sm-**L1**/(Sm-**L1**)^•–^ = –1.50 V) and Sm-**L2** (*E*_p_(Sm-**L2**/(Sm-**L2**)^•–^ = −1.40 V), suggesting that the electron transfer from the photoexcited coumarin ligand to the Sm(III) center is favorable.

The reactions were performed using 10 mol % of the Sm-**L1** or Sm-**L2** complex under irradiation with 40 W 463 nm LEDs. Zinc metal (conditions A) or *N*,*N*-diisopropylethylamine (DIPEA, conditions B) were used as the sacrificial co-reductants. Under these conditions, the dimerization of benzyl chloride and dehalogenation of aryl bromides were achieved (Figure 3b), as well as the reduction of benzophenone imine, intramolecular pinacol coupling, and reductive pyrazine synthesis. By contrast, no reaction was observed when the corresponding Gd complex was used instead of the Sm complex in the dimerization of benzyl chloride. Although Gd(III) has a similar ionic radius and Lewis acidity to Sm(III), it is a redox-inert ion with a [Xe]4f^7^ electron configuration, and its reduction is highly unfavorable (*E*(Gd(III)/Gd(II)) < −3.5 V vs SHE) [78]. These results indicated that the excited coumarin ligand did not directly reduce the substrate; instead, the photogenerated Sm(II) species served as a catalytically active reductant.

**Figure 4:**
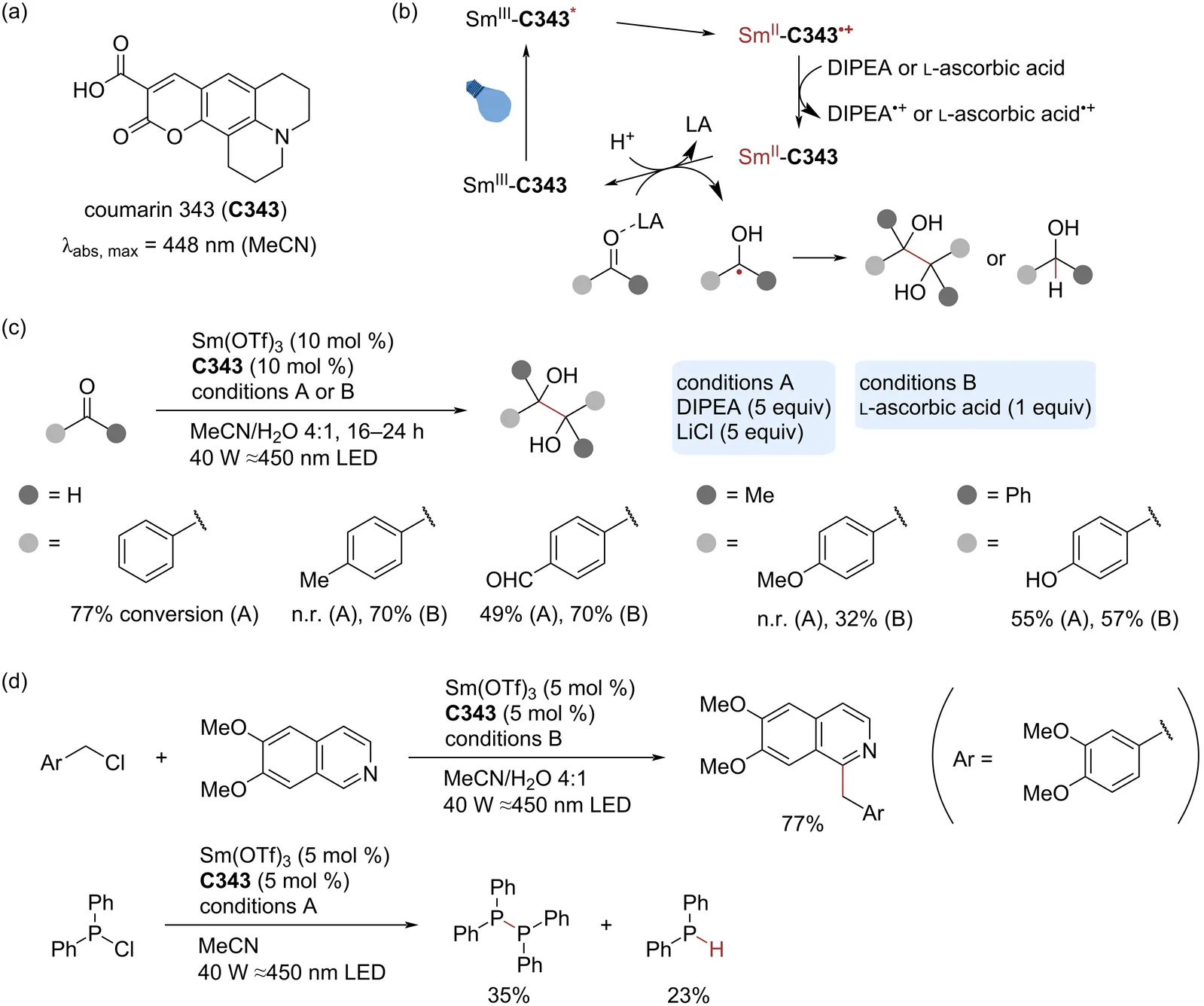
Commercially available coumarin 343 (**C343**) as a photocatalyst [66]. (a) Structure of **C343**. (b) Proposed reaction mechanism. LA = Lewis acid. (c) Pinacol coupling reactions. (d) Selected examples of other synthetic applications.

In 2024, Borbas et al. reported an Sm(III)/Sm(II) photocatalytic system utilizing the commercially available dye coumarin 343 (**C343**) (Figure 4a) [66]. **C343** exhibits a strong absorption band centered at approximately 450 nm, enabling efficient photoexcitation under irradiation with 40 W ca. 450 nm LEDs. Pinacol coupling of benzaldehyde was investigated as a model reaction (Figure 4c). The Sm(OTf)_3_/**C343** system afforded 77% conversion to the corresponding pinacol product using DIPEA as a co-reductant in MeCN/H_2_O (conditions A). The replacement of Sm(OTf)_3_ with the redox-inactive Gd(OTf)_3_ resulted in 7% conversion to the product. Furthermore, the conversion decreased to less than 1% in the dark and to 4% in the absence of Sm(OTf)_3_, suggesting that the photochemical Sm(II) formation is important for reactivity. In addition to DIPEA, ʟ-ascorbic acid was examined as a co-reductant (conditions B) (Figure 4c and d). Under these conditions, the Sm/**C343** catalytic system was applicable to the pinacol coupling of aromatic aldehydes and ketones, dehalogenative coupling of benzyl chloride, and reduction of azobenzene and diphenylphosphine chloride.

The proposed reaction mechanism involves a single-electron transfer from the photoexcited **C343** (C343*) to the Sm(III) center which generates Sm(II)-**C343^•+^** (Figure 4b). Subsequently, **C343^•+^** is reduced by either DIPEA (*E*_ox_ = 0.86 V vs SCE) or ʟ-ascorbic acid (*E*_ox_ = 0.35 V vs SHE), generating Sm(II)-**C343**, which serves as the active reducing species. Sm(II)-**C343** then reduces carbonyl compounds via single-electron transfer to generate ketyl radicals, while regenerating Sm(III)-**C343**. The authors also considered an alternative pathway involving the initial single-electron reduction of **C343** to generate Sm(III)-**C343^•−^**. However, no changes in the fluorescence intensity or lifetime of **C343** were observed upon increasing the concentration of ʟ-ascorbic acid in the presence of Gd(III), indicating that this pathway is unlikely. In addition, the Sm(III)–OR species generated after substrate reduction are often difficult to reduce; therefore, the addition of a proton source [15,23,38] or silylating reagents [16–17,19–21,31,35–37] has been reported to maintain the Sm(III)/Sm(II) catalytic cycle in conventional catalytic approaches. Furthermore, Sm(OR)_3_ species are known to be redox-inactive [15]. In contrast, the Sm-**C343** system was employed in MeCN/H_2_O, where Sm(III)–OH species, such as Sm(OH)_3_, may form during the reaction. Although the actual formation of these species and their effects on the regeneration of Sm(II) remain unclear, it is noteworthy that water is tolerated as a co-solvent.

In 2025, Borbas et al. systematically evaluated the photophysical properties and photocatalytic activities of a series of organic dyes including coumarin, carbostyril, and rhodamine derivatives [67]. In this study, mixtures with Eu(OTf)_3_ were investigated, although Sm(OTf)_3_ was also examined in selected cases. Notably, **C343** and 7-hydroxy-4-methoxymethylcoumarin promote the reductive dimerization of benzyl chloride, demonstrating their ability to construct a photochemical Sm(III)/Sm(II) redox cycle. These results suggest that simple catalytic systems based on commercially available dyes can serve as practical platforms for photochemical Sm(III)/Sm(II) catalysis.

**Figure 5:**
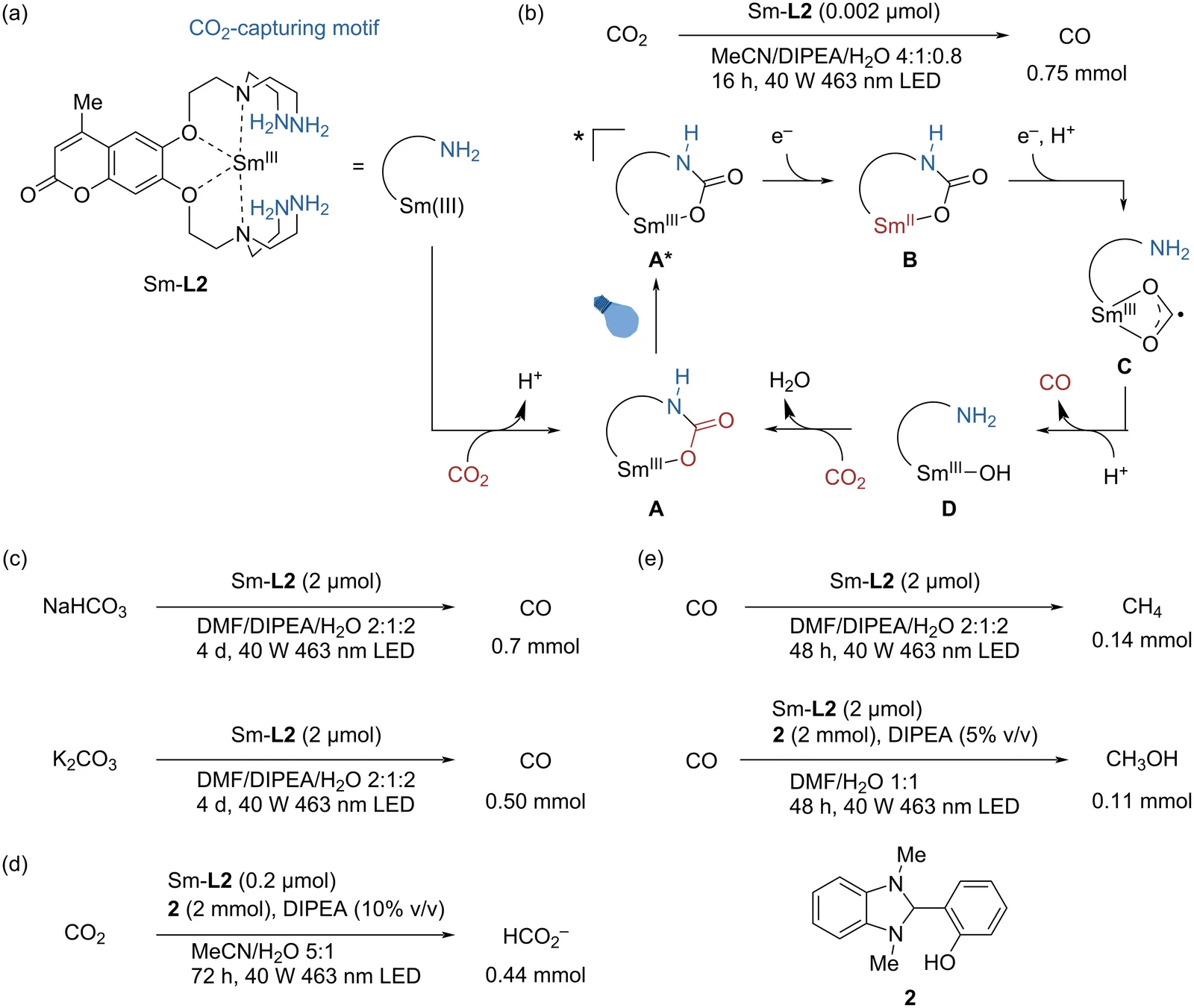
Reduction of CO_2_, CO, and (bi)carbonates [68]. (a) Design of the Sm-**L2** catalyst. (b) Reduction of CO_2_ to CO. (c) Reduction of (bi)carbonates to CO. (d) Reduction of CO_2_ to HCO_2_^−^. (e) Reduction of CO to methane and methanol.

In the same year, Borbas and colleagues extended the Sm(III)/Sm(II) catalysis to the reduction of small molecules including CO_2_, bicarbonate, carbonate, and CO [68]. The homogeneous catalyst Sm-**L2** is a multifunctional complex comprising a 6,7-dioxycoumarin moiety for light absorption and photoredox activity, a polyamine unit for CO_2_ capture through carbamate formation, and a samarium redox center (Figure 5a). Under a CO_2_ atmosphere, Sm-**L2** catalyzed the formation of CO in a MeCN/DIPEA/H_2_O mixed solvent under irradiation with 40 W 463 nm LEDs (Figure 5b). When the catalyst loading was reduced to 0.002 μmol, the turnover number (TON) for CO formation reached 378,000. According to the proposed reaction mechanism, the primary amine of **L2** first reacts with CO_2_ to form an **L2**-carbamate species, as confirmed by ^13^C NMR and FTIR spectroscopy. Upon photoexcitation of Sm(III)-**L2** carbamate **A**, electron transfer from the excited coumarin moiety to the Sm(III) center, followed by single-electron reduction by DIPEA, generates Sm(II)-**L2** carbamate **B**. Subsequently, proton-coupled electron transfer affords the CO_2_ radical anion **C**, which undergoes C–O-bond cleavage to release CO and generates Sm(III) hydroxide species **D**. Intermediate **D** reacts with another CO_2_ molecule to regenerate **A** with the concomitant formation of water.

The authors also reported the reduction of bicarbonates and carbonates (Figure 5c). Sm-**L2** enables direct CO formation from a broad range of (bi)carbonate substrates, including NaHCO_3_, KHCO_3_, Na_2_CO_3_, K_2_CO_3_, and CaCO_3_. Furthermore, the product selectivity can be switched by changing the reaction conditions. When DIPEA was used as the co-reductant under a CO_2_ atmosphere, CO was produced selectively (>99%), whereas the addition of hydrogenated benzimidazole derivative **2** switched the selectivity to formate (>99%) (Figure 5d). Radical cation **2**^•+^ acts as an efficient hydrogen atom donor, thereby favoring hydrogen atom transfer over C–O-bond cleavage of **C**. In addition, methane was selectively produced using DIPEA under a CO atmosphere, whereas methanol was selectively obtained in the presence of both DIPEA and **2** (Figure 5e).

**Figure 6:**
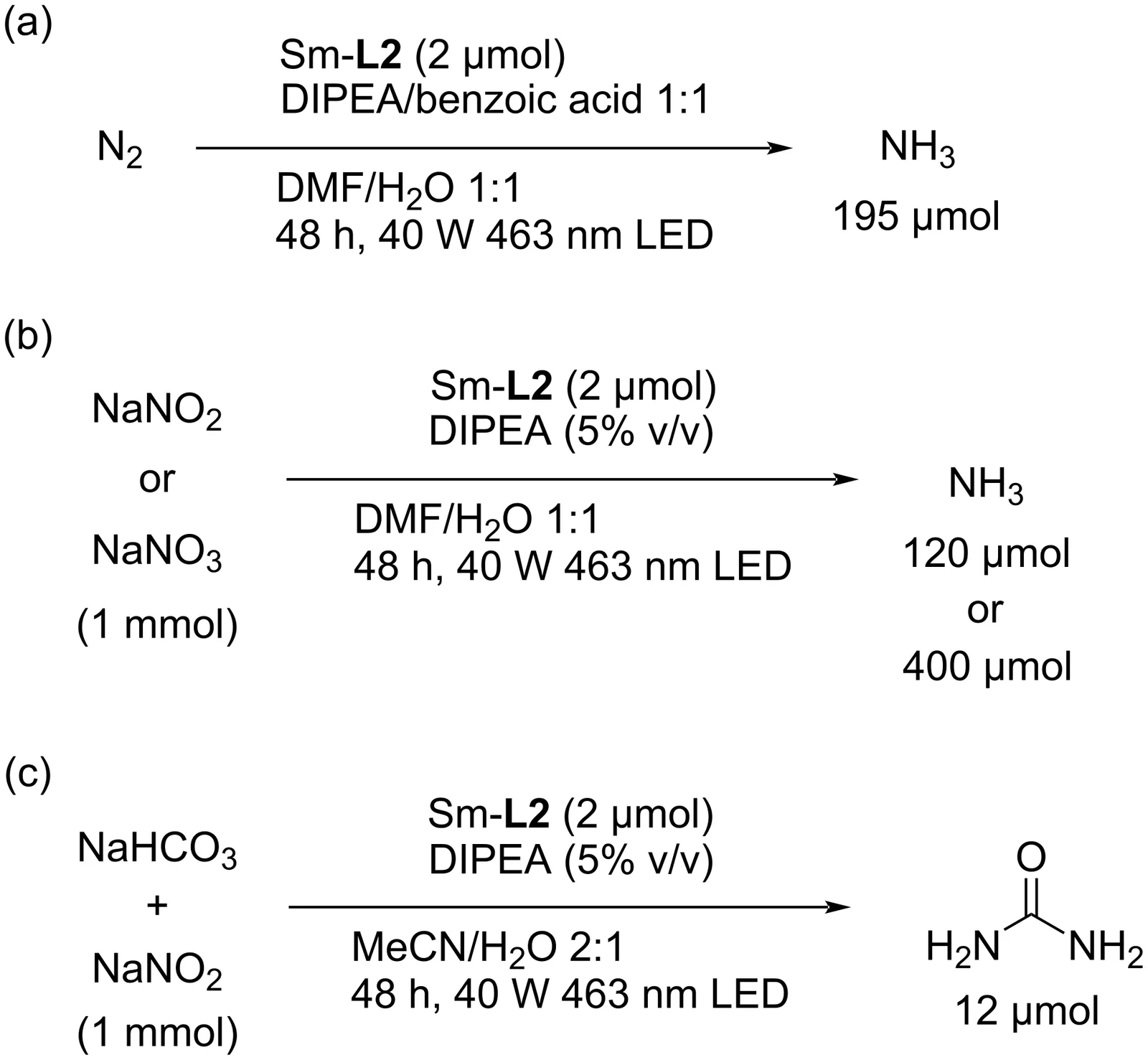
Synthesis of ammonia and urea [69]. Ammonia production from (a) N_2_ and (b) NaNO_2_ and NaNO_3_. (c) Urea formation from NaHCO_3_ and NaNO_2_.

Also in 2025, Borbas et al. also reported the synthesis of ammonia from dinitrogen (N_2_), nitrite (NO_2_^−^), and nitrate (NO_3_^−^) [69]. Upon irradiation with 40 W 463 nm LEDs, the Sm-**L2** complex achieved a TON of 98 for NH_3_ formation under an N_2_ atmosphere using DIPEA and benzoic acid (Figure 6a). Sequential electron- and proton-transfer processes mediated by photogenerated Sm(II)-**L2** or Sm(II)-**L2**^•−^ drive the catalytic multielectron reduction of N_2_. The catalytic system was also effective in the reduction of nitrogen oxides; NaNO_2_ and NaNO_3_ were converted to ammonia with TONs of 60 and 200, respectively (Figure 6b). Furthermore, the authors stated that the cooperative reduction of bicarbonate and nitrite enabled urea synthesis with a TON of 6 (Figure 6c). Overall, Sm-**L2** functions as a versatile photoreductive catalyst capable of reducing CO_2_, bicarbonate, carbonate, CO, N_2_, nitrite, and nitrate, as well as enabling urea synthesis, highlighting the potential of photo-driven Sm(III)/Sm(II) catalysis for carbon and nitrogen cycling.

### 9,10-Diphenylanthracene-based antenna ligand

**Figure 7:**
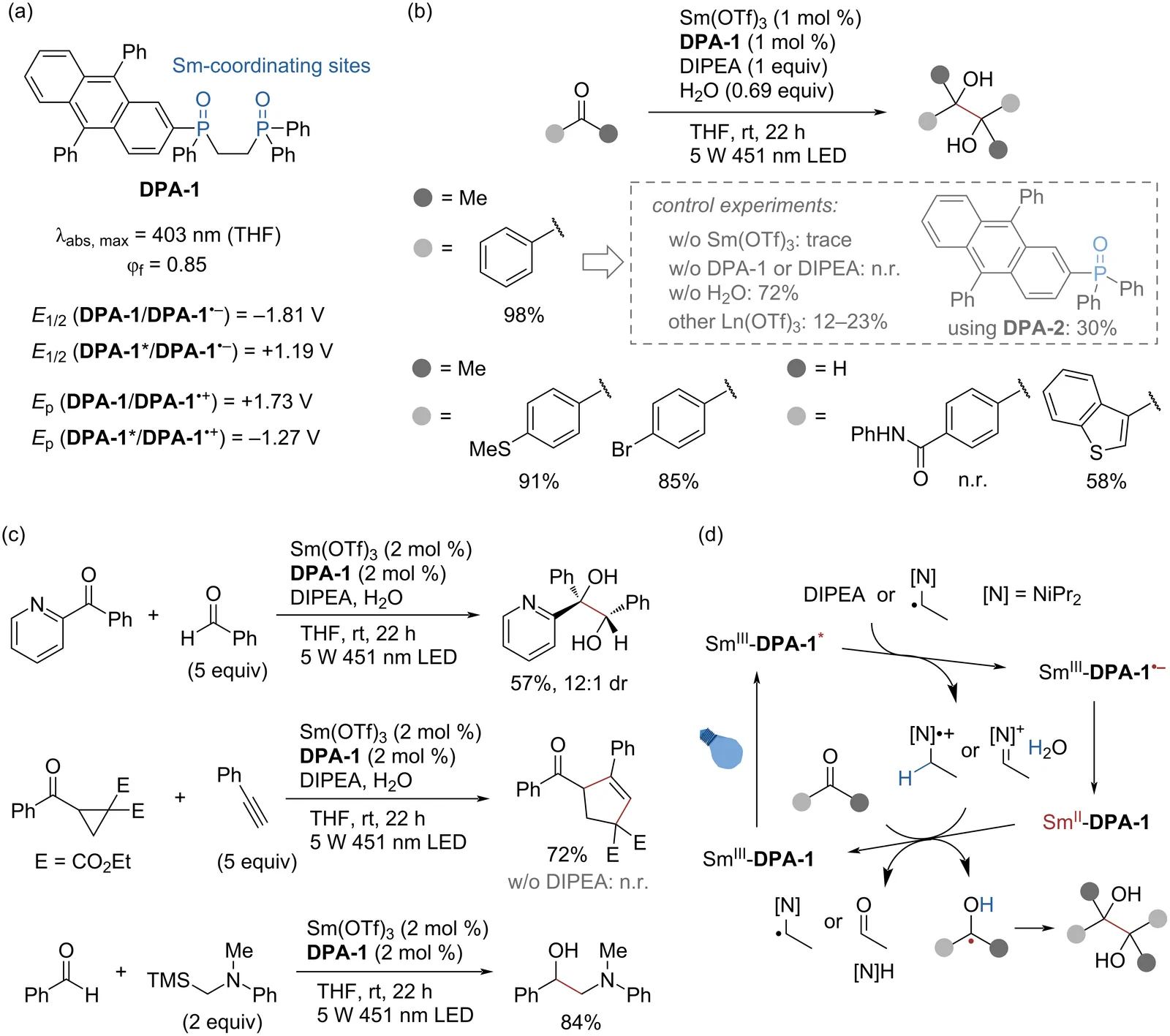
9,10-Diphenylanthracene-based bidentate phosphine oxide **DPA-1** for samarium-catalyzed reductive transformations [70]. (a) Design and properties of **DPA-1**. Potentials are shown vs SCE in THF. (b) Pinacol couplings and control experiments. (c) Other selected examples. (d) Proposed reaction mechanism.

In 2024, Kuribara and Nemoto et al. developed a visible-light-absorbing antenna ligand, **DPA-1**, and reported reductive transformations based on a photo-driven Sm(III)/Sm(II) catalytic system at low catalyst loadings [70]. The multifunctional ligand **DPA-1** was designed by integrating a 9,10-diphenylanthracene (**DPA**) chromophore with bidentate phosphine oxide units (Figure 7a). **DPA** is known to exhibit a high fluorescence quantum yield and a highly reducing excited state under visible-light irradiation [79]. In addition, phosphine oxide is stable under reducing conditions and exhibits a high affinity for hard Lewis acidic samarium ions, allowing **DPA-1** to function as a redox-active antenna ligand for samarium.

Pinacol coupling of acetophenone was first examined using 1 mol % Sm(OTf)_3_ and **DPA-1** [70]. The corresponding 1,2-diol was obtained in 98% yield in the presence of DIPEA (1 equiv) and water (0.69 equiv) under irradiation with 5 W 451 nm LEDs (Figure 7b). Control experiments confirmed that Sm(OTf)_3_, **DPA-1**, DIPEA, and light irradiation were essential for the reaction. The use of other Ln(OTf)_3_ salts resulted in significantly lower yields, suggesting that the efficient generation of Sm(II), followed by inner-sphere electron transfer, plays a more important role than the substrate activation by Ln(III) as a Lewis acid. Moreover, replacing **DPA-1** with the monodentate analog **DPA-2** decreased the yield to 30%, highlighting the importance of the bidentate phosphine oxide unit for high catalytic activity. Fluorescence quenching experiments supported the fact that **DPA-1** coordinates more strongly with Sm(III) than with **DPA-2**, thereby favoring the formation of a 1:1 complex. The substrate scope included a variety of aromatic ketones and aldehydes; in most cases, the pinacol coupling proceeded with only 1 mol % of the catalyst. Conversely, the amide substrate exhibited no reactivity, presumably because a competitive coordination of the amide group to the Sm center inhibits the coordination and subsequent inner-sphere single-electron reduction of the reactive carbonyl groups. The Sm-**DPA-1** system was also applicable to the cross-pinacol coupling of 2-pyridyl ketones with aldehydes, as well as to a variety of reductive transformations, including flavone dimerization, C–O-bond cleavage of epoxides, C–C-bond cleavage of cyclopropanes, and ketyl–olefin coupling (Figure 7c). In addition, radical coupling between aromatic aldehydes and α-silylamines was achieved through a combination of single-electron reduction and oxidation reactions.

According to the proposed reaction mechanism, photoexcited **DPA-1** (*E*_1/2_(DPA-1*/**DPA-1^•−^**) = +1.19 V vs SCE) is proposed to be reduced by DIPEA (*E*_p_ = +1.11 V vs SCE) to generate **DPA-1^•−^** (*E*_1/2_(**DPA-1**/**DPA-1^•−^**) = −1.81 V vs SCE), which subsequently transfers an electron to the coordinated Sm(OTf)_3_ (*E*_p_ = −1.19 V vs SCE) to afford Sm(II)-**DPA-1** (Figure 7d). This proposed mechanism is supported by the observed quenching of **DPA-1** in the presence of DIPEA. In addition, the single-electron reduction of the cyclopropyl ketone, followed by ring opening of the cyclopropane moiety, does not proceed in the absence of DIPEA, further suggesting the involvement of **DPA-1^•−^** (Figure 7c). In contrast, in the system reported by Borbas et al. [65–69], Sm(II) formation is proposed to occur via single-electron transfer from the excited coumarin-based photocatalyst to Sm(III), indicating a different sequence of electron-transfer events between the two systems. The resulting Sm(II)-**DPA-1** species reduces aromatic carbonyl compounds via single-electron transfer to generate ketyl radicals, which subsequently undergo protonation and radical coupling to afford the corresponding 1,2-diols. A small amount of water was required to hydrolyze the iminium cation generated by the oxidation of DIPEA, while also serving as an additional proton source.

Among the aforementioned photochemical Sm(II) catalytic systems, a common feature of the catalyst design is the close proximity between the photoredox-active site and the Sm center. The coumarin-based photocatalyst reported by Borbas et al. are either incorporated into the Sm complexes or coordinated to Sm(III) [65–69]. In addition, the bidentate phosphine oxide ligand **DPA-1** developed by Kuribara and Nemoto et al. coordinates strongly with Sm(III), which contributes to the high catalytic activity [70]. Such proximity effects between reactive centers have long been recognized as an important concept for enhancing reaction rates [80–81]. These findings are also relevant to the design of the present catalytic system. The close spatial arrangement may facilitate the catalytic process by promoting photoinduced electron transfer and thus represents an important design consideration for efficient photoinduced Sm(III)/Sm(II) redox catalysis.

**Figure 8:**
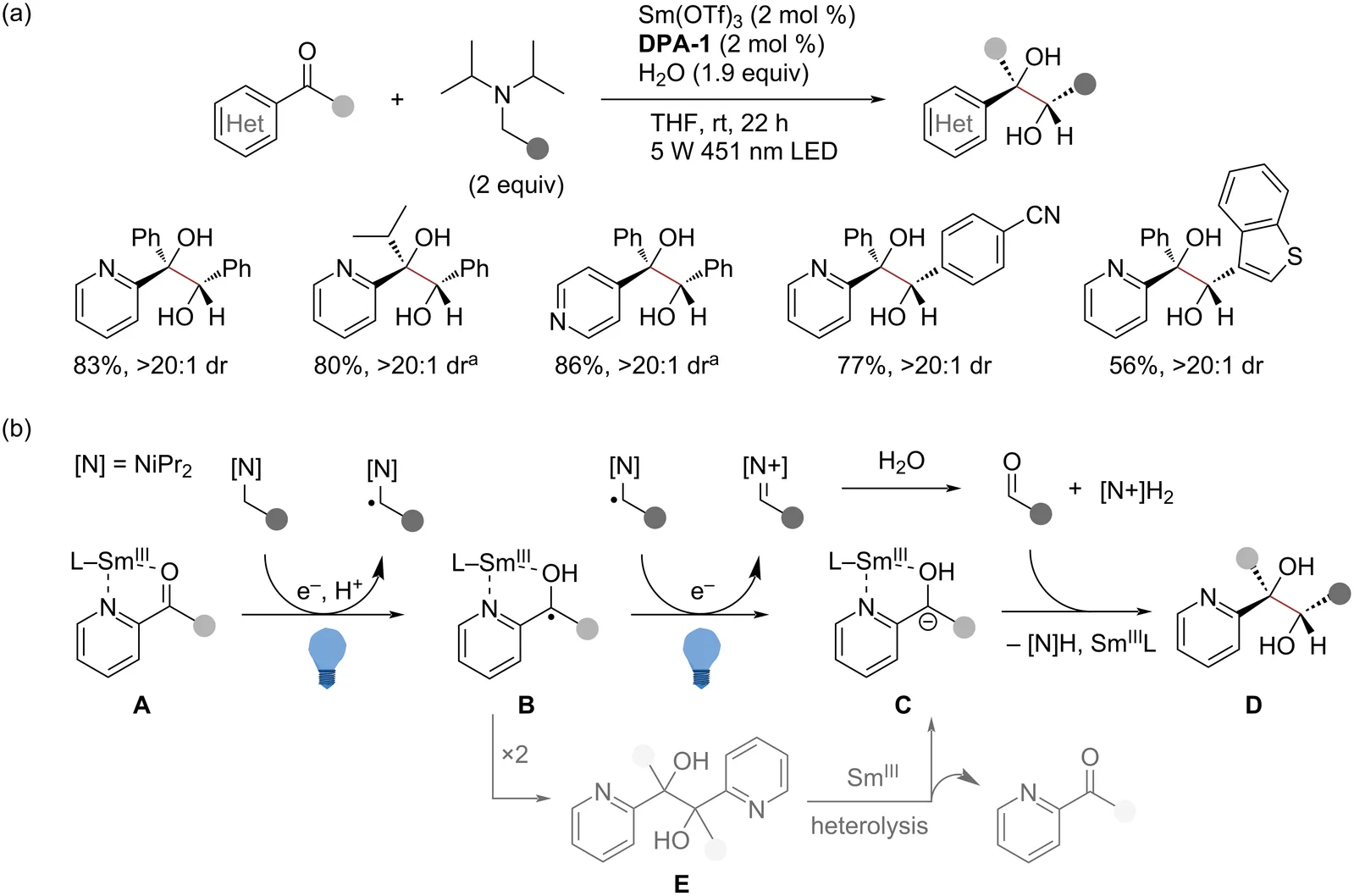
Redox-neutral cross-pinacol coupling [71]. (a) Selected examples. (b) Proposed reaction mechanism. ^a^Sm(OTf)_3_ and **DPA-1** (5 mol %) were used.

In 2026, Kuribara and Nemoto et al. extended their previous cross-pinacol coupling to a redox-neutral variant, enabling the cross-selective and highly diastereoselective C–C-bond formation without the need for an external reductant [71]. Using Sm(OTf)_3_ and **DPA-1** under irradiation with 5 W 451 nm LEDs, pyridyl ketones were reacted with aldehydes generated by the oxidation of benzylamine derivatives followed by hydrolysis to afford the corresponding cross-coupling products in up to 86% yield with excellent diastereoselectivity (>20:1 dr) (Figure 8a). The substrate scope included 2-pyridyl and 4-pyridyl ketones as well as a variety of benzylamine derivatives, demonstrating a broad substrate scope.

According to the proposed mechanism, the photoexcited antenna ligand, **DPA-1**, reduces Sm-coordinated 2-pyridyl ketone **A** to generate ketyl radical **B** (Figure 8b). The second photoreduction enables a radical–polar crossover to afford Sm-coordinated carbanion **C**. Unlike conventional pinacol coupling reactions, this strategy suppresses uncontrolled radical coupling by exploiting a polar reaction pathway. During reduction, the amine is oxidized and hydrolyzed to the corresponding aldehyde. Finally, nucleophilic addition of **C** to the aldehyde afforded the cross-coupling product **D**. Time-course NMR studies and control experiments further revealed that the homopinacol side product **E** underwent heterolytic C–C-bond cleavage to afford the 2-pyridyl ketone and the reactive species **C** in the presence of Sm(OTf)_3_, thereby improving the cross-selectivity. Overall, this study integrates three catalytic features into a single reaction system: radical–polar crossover enabled by two sequential single-electron reductions, in situ generation of aldehydes through photooxidation, and Sm(III)-mediated C–C-bond heterolysis via Lewis acid catalysis. This multifunctional strategy expands the scope of conventional Sm(II)-mediated reduction chemistry and enhances the development of versatile photo-driven samarium-catalyzed transformations.

### Hantzsch ester and iridium photocatalyst

**Figure 9:**
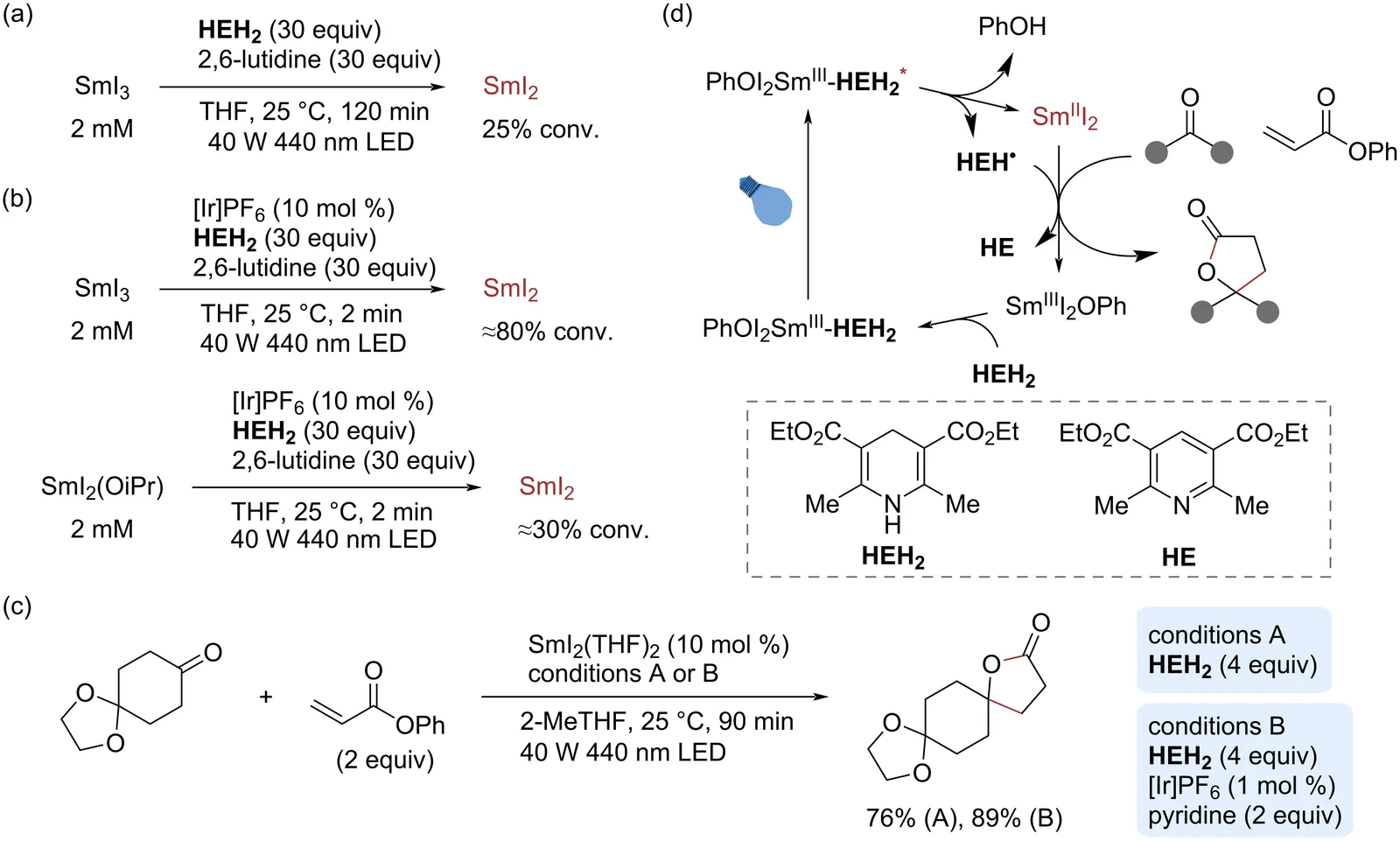
Photo-driven generation of SmI_2_ and reductive lactone formation [72]. (a) SmI_3_ reduction by Hantzsch ester (**HEH_2_**). (b) SmI_3_ and SmI_2_(OiPr) reduction by [Ir(ppy)_2_(dtbbpy)]PF_6_ (= [Ir]PF_6_) and **HEH_2_**. (c) Reductive lactone formation. (d) Proposed reaction mechanism (conditions A).

In 2024, Peters et al. reported two methods for generating Sm(II): one using a stoichiometric amount of Hantzsch ester (**HEH_2_**) as a photoreductant and the other combining **HEH_2_** with an Ir photocatalyst [72]. The **HEH_2_** functions as a multifunctional photoreductant; its ester groups coordinate to samarium, and upon irradiation with 40 W 440 nm LEDs, the photoexcited **HEH_2_** acts as a potent single-electron reductant (Figure 9a). Importantly, the authors quantitatively determined the amount of SmI_2_ using UV–vis absorption spectroscopy. SmI_2_ was generated from SmI_3_ with 25% conversion in the presence of 30 equiv of **HEH_2_** and 2,6-lutidine.

To improve the efficiency of Sm(III) photoreduction, an iridium complex, [Ir(dtbbpy)(ppy)_2_]PF_6_ was introduced as a photocatalyst (Figure 9b). Upon visible-light irradiation, the photoexcited Ir(III) complex underwent reductive quenching by **HEH_2_** to generate the corresponding Ir(II) species, which rapidly reduced Sm(III) to Sm(II). Consequently, SmI_2_ was generated from SmI_3_ with approximately 80% conversion within 2 min. Furthermore, the authors investigated the photoreduction of the samarium alkoxide SmI_2_(OiPr), and SmI_2_ was generated with approximately 30% conversion. This transformation proceeds through cooperative single-electron transfer from the Ir(II) species and proton transfer from **HEH_2_^•+^**. Moreover, the photo-driven generation of Sm(II) was demonstrated in the presence of various coordinating ligands, including ethylene glycol, chiral amino diols, bromide, HMPA, and phosphine oxides. In this study, the reduction of Sm(III) to Sm(II) was shown to proceed not only with the Hantzsch ester alone but also with an Ir-based external photocatalyst and Hantzsch ester, thereby providing greater flexibility in the design of Sm-catalyzed reduction reactions.

The established reaction conditions were subsequently applied to the reductive lactonization of ketones with acrylates (Figure 9c). Under irradiation with 40 W 440 nm LEDs, the corresponding lactones were obtained in 76% yield using **HEH_2_** alone (conditions A) and in 89% yield using the Ir photocatalyst (conditions B). According to the proposed mechanism, **HEH_2_** that is coordinated to the Sm(III) species is photoexcited and undergoes a single-electron transfer to generate SmI_2_ (Figure 9d). The resulting SmI_2_ promotes reductive coupling between the ketone and the acrylate, and the resulting carbon-centered radical undergoes hydrogen atom transfer from the Hantzsch ester radical (**HEH**^•^), followed by cyclization to afford the corresponding lactone. The resulting Sm(III)-OPh species underwent photochemical reduction and protonation by **HEH_2_**-derived protons to regenerate SmI_2_. This study demonstrates that **HEH_2_**, which is less susceptible to back-electron transfer, serves as an effective photoreductant for the generation of SmI_2_. These findings provide direct experimental evidence for the photochemical reduction of Sm(III) and establish a practical platform for photo-driven Sm(II) catalysis.

In 2026, Reisman and Peters et al. reported a samarium-catalyzed asymmetric reduction reaction [73]. Conventional methods require stoichiometric amounts of the Sm(II) reductants and chiral ligands [3,82]. To overcome this limitation, the authors developed a catalytic asymmetric reduction reaction based on the Sm(III)/Sm(II) redox cycle. However, subsequent mechanistic studies revealed that the reaction also proceeds in the presence of redox-inert Gd(III), indicating that it operates through a distinct mechanism. Nevertheless, this study provides important mechanistic insights for the future development of samarium-catalyzed asymmetric reductions.

**Figure 10:**
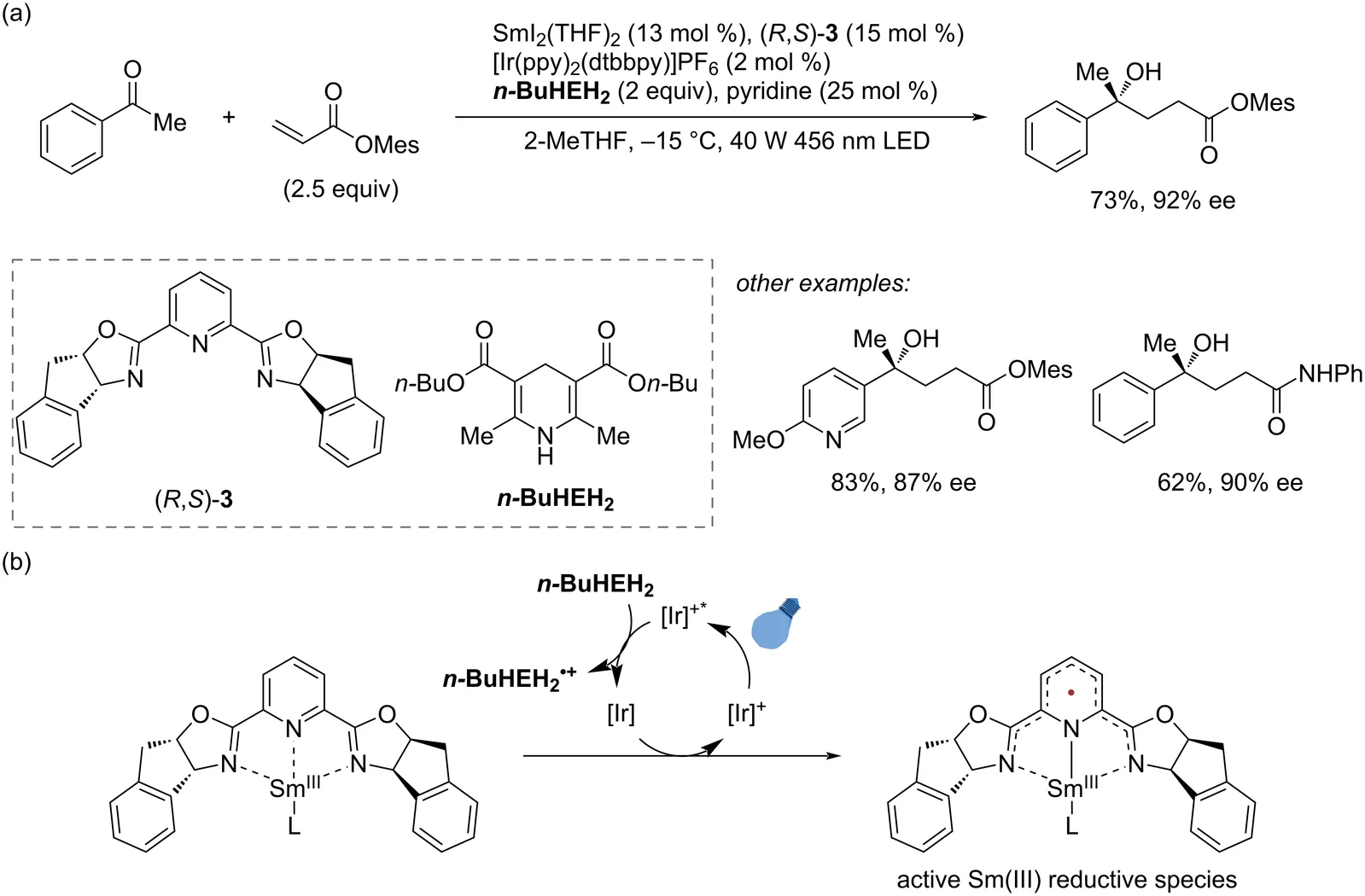
Samarium-catalyzed asymmetric ketyl–olefin coupling [73]. (a) Optimized conditions and selective examples. Mes = mesityl. (b) Actual Sm(III) reductive species.

The authors developed an asymmetric ketyl–olefin coupling of aromatic ketones with acrylates by combining an SmI_2_/PyBOX chiral ligand complex with an iridium photocatalyst and ***n*-BuHEH_2_**, affording the corresponding products in up to 98% ee (Figure 10a). Mechanistic studies revealed that the catalytically active species was not the initially proposed Sm(II) complex but rather an Sm(III)-stabilized PyBOX radical anion complex (Figure 10b). UV–vis absorption spectroscopy showed that the characteristic absorption band of SmI_2_ disappeared upon the addition of the PyBOX ligand, whereas a new absorption band emerged in the visible region. The same absorption band was also observed under the catalytic conditions using SmI_3_, the Ir photocatalyst, and Hantzsch ester upon irradiation with 40 W 456 nm LEDs. Electrochemical measurements and DFT calculations indicated that the unpaired electron was localized on the ligand rather than on the samarium center. Furthermore, electron transfer from the Sm-PyBOX radical complex to the ketone was thermodynamically favorable, supporting its role in ketyl radical generation. These results indicated that the Sm–PyBOX radical complex was formed via single-electron transfer from the Ir(II) species and served as the actual reductive species.

## Conclusion

This review summarize the recent progress in photochemical strategies for Sm(II) catalysis. The development of samarium complexes bearing photosensitizing units, visible-light-absorbing redox-active antenna ligands, and dual catalytic systems combining samarium catalysts with external photocatalysts has enabled a wide range of catalytic transformations involving Sm(II) species. These photochemical strategies enhance the sustainability of samarium catalysis and substantially expand its synthetic utility. These systems provide high reducing power and chemoselectivity and enable new reaction designs through the integration of Sm(II) catalysis with photoredox oxidation and Lewis acid catalysis. However, these studies have also revealed that not all transformations proceed through Sm(II) as an active reducing species. Nevertheless, the catalytic generation of Sm(II) remains crucial for efficient inner-sphere electron transfer, contributing to improved reaction efficiency and chemoselectivity.

Future challenges include the development of more efficient catalytic systems with lower catalyst loadings and faster catalytic turnover, expansion of the reaction and substrate scope, realization of catalytic asymmetric reductions based on the Sm(III)/Sm(II) redox cycle, and further exploration of the photochemical activation of Sm(II) species generated in situ and strongly reducing other divalent lanthanoids. Photochemical strategies for Sm(II) catalysis represent a rapidly evolving field at the intersection of lanthanoid chemistry, photochemistry, and radical chemistry, and are thus expected to provide a versatile platform for sustainable and efficient reductive transformations.

## Data Availability

Data sharing is not applicable as no new data was generated or analyzed in this study.
